# ADHD and metabolic syndrome: behavioral and weight-related pathways to cardiovascular risk

**DOI:** 10.1007/s00406-026-02246-6

**Published:** 2026-05-19

**Authors:** Georg C. Ziegler, Katharina Kürten, Karla Roshop, Matthias Nieberler, Bodo Warrings, Sarah Kittel-Schneider, Klaus-Peter Lesch, Martin J. Herrmann, Stefan Störk, Jürgen Deckert

**Affiliations:** 1 Department of Psychiatry, Psychosomatics and Psychotherapy, Center of Mental Health, University Hospital of Würzburg, Würzburg, Germany; 2 Department of Psychiatry and Neurobehavioural Science, University College Cork, Cork, Ireland; 3 Department of Psychiatry and Psychotherapy, University Hospital Erlangen, Friedrich-Alexander-University, Erlangen-Nuremberg, Germany; 4 Department of Child- and Adolescent Psychiatry, Psychosomatics and Psychotherapy, Center of Mental Health, University of Würzburg, Würzburg, Germany; 5 Department of Psychiatry and Neuropsychology, School for Mental Health and Neuroscience (MHeNs), Maastricht University, Maastricht, The Netherlands; 6 Comprehensive Heart Failure Center (CHFC), University Hospital of Würzburg, Würzburg, Germany; 7 Department of Internal Medicine I, University Hospital of Würzburg, Würzburg, Germany; 8 Institute of Clinical Epidemiology and Biometry, University of Würzburg, Würzburg, Germany

**Keywords:** ADHD, Metabolic syndrome, Cardiovascular risk, Impulsivity, Eating behavior, Obesity

## Abstract

**Background:**

Attention-deficit/hyperactivity disorder (ADHD) has been linked to obesity and type 2 diabetes, suggesting heightened cardiovascular risk. Whether adults with ADHD also show increased rates of metabolic syndrome (MetS), a cluster of central obesity, dyslipidemia, impaired glucose regulation, and hypertension, remains unclear.

**Methods:**

We examined a young to middle-aged adult cohort of 83 patients and 82 healthy controls (HC). The primary objective was to test whether MetS (Joint Interim Statement criteria) was more prevalent in ADHD than in HC. To explore potential pathways linking ADHD with cardiovascular risk, we additionally assessed anthropometric indices, 24-h blood pressure (BP), heart rate (HR), and inflammatory markers (interleukin-6, C-reactive protein). Finally, we fitted a serial mediation model examining whether hyperactivity/impulsivity (HI), eating behavior, and body mass index (BMI) mediated the association between ADHD and BP.

**Results:**

A higher proportion of ADHD patients fulfilled MetS criteria (OR 2.29). Adults with ADHD showed higher waist circumference, BMI, and overweight/obesity rates but did not differ in glucose and blood lipid levels. Non-stimulant medication and psychiatric comorbidity were more common among patients with MetS. Inflammatory markers were unrelated to ADHD. However, ADHD patients demonstrated slightly higher mean 24-h HR and nighttime systolic BP. The association between ADHD and nighttime systolic BP was mediated by obesity as a MetS constituent, and by HI and disordered eating as behavioral risk factors.

**Conclusion:**

Young to middle-aged adults with ADHD displayed increased MetS prevalence and subtle elevations in nighttime BP and HR, suggesting an early cardiovascular risk trajectory. Behavioral factors, particularly overeating and weight gain, and psychiatric comorbidity appear to contribute to this risk. These findings support early cardiovascular screening and weight-management interventions in adults with ADHD.

**Supplementary Information:**

The online version contains supplementary material available at 10.1007/s00406-026-02246-6.

## Introduction

Attention-deficit/hyperactivity disorder (ADHD) is characterized by an impaired focus of attention, increased impulsivity, and motor hyperactivity/inner restlessness, regularly accompanied by emotional dysregulation [1, 2]. ADHD frequently persists into adulthood, with more than half of those diagnosed in childhood continuing to experience impairing symptoms [3]. With a high rate of comorbidity, ADHD is considered a neurodevelopmental precursor disorder for other psychiatric conditions, including substance use disorders (SUD) [4], and depressive disorders [5]. Such comorbidity increases disease burden, impairs psychosocial and neuropsychological functioning [6], and elevates risk for suicide attempts [7].

Large registry studies have shown that ADHD is associated with reduced life expectancy [8–10]. Excess mortality is mainly driven by unnatural causes, particularly accidents [8, 9]. Stimulant medication reduces accident risk, supporting a link between ADHD symptoms and risky behaviors including careless driving [11–13]. Psychiatric comorbidity further contributes to reduced life expectancy, with childhood-onset comorbidity linked to deaths from natural causes [9]. Hence, patients with ADHD may also be at risk for reduced life expectancy due to somatic comorbidity.

Mental and somatic diseases often interact. Depressive disorders worsen cardiovascular outcomes [14], while cardiovascular disease increases the risk of developing depression [15]. Beyond psychiatric comorbidity, several somatic conditions are more prevalent among individuals with ADHD, including obesity, asthma, migraine, and celiac disease [16]. The association with obesity has been replicated in large registry studies [17, 18]. Earlier work did not consistently link ADHD to cardiovascular disease, as noted in the systematic review by Instanes et al. [16], but recent evidence indicates a higher cardiovascular disease burden in ADHD [19]. Large registry studies showed that adults with ADHD are at increased risk for type 2 diabetes, with odds ratios around 2 [20–22], and appear to develop cardiovascular disease earlier in life than their peers [23], suggesting a dual burden of psychiatric and somatic morbidity from an early age.

Cardiovascular risk arises from genetic [24] and behavioral factors [25], that contribute to autonomic hyperarousal, tissue inflammation, and atherogenesis [26]. Metabolic syndrome (MetS), defined by central obesity, elevated fasting glucose or insulin resistance, dyslipidemia, and elevated blood pressure (BP), is an early predictor of cardiovascular disease and mortality [27]. Earlier onset of MetS confers greater risk than later-onset MetS [28]. Despite consistent evidence linking ADHD to obesity, only one small study has assessed MetS prevalence in adults with ADHD [29].

Against the background of emerging associations between ADHD and cardiovascular disease, and indications of an early cardiovascular risk trajectory [23], we tested whether MetS is more common in apparently healthy young to middle-aged adults with ADHD compared with age-matched healthy controls (HC). We additionally examined psychopathological and behavioral variables, anthropometric measurements, inflammatory markers, and 24-hour BP and heart rate (HR) regulation, to identify potential pathways linking ADHD with elevated cardiovascular risk.

## Methods

### Recruitment strategy

Study participants were recruited for the CoR-ADHD study between November 2022 and July 2025. This cross-sectional case-control study investigates cardiovascular regulation and risk factors in adults with ADHD. Procedures were approved by the ethics committee of the University Hospital of Würzburg (no. 272/20), Germany, and conducted in accordance with the Declaration of Helsinki (WMA, 2013). ADHD patients were partly recruited from our specialized outpatient unit (*n* = 23), and additional patients (*n* = 60) and HC (*n* = 82) were enrolled through press releases, newspaper and online advertisements, flyers, newsletters, and the intranet of the University Hospital Würzburg. Externally diagnosed patients were interviewed by an experienced psychiatrist (G.C.Z.), and medical records were reviewed to verify diagnostic plausibility.

### Selection criteria for study participants

Eligible participants were 18 to 65 years old and able to provide informed consent. Exclusion criteria were known arterial hypertension, heart failure, or use of antihypertensive/cardiac medication (e.g., beta-blockers, ACE inhibitors, AT1 blockers, renin antagonists, calcium antagonists, diuretics). Patients on psychostimulant medication had to pause medication one week prior to study assessments to avoid acute medication effects on cardiovascular measurements. Patients prescribed medical cannabis by their private practice psychiatrist had to pause use likewise. Individuals with neurological disorders (e.g., stroke, epilepsy, multiple sclerosis, Parkinson’s disease, dementia) or acute SUD were excluded. HC had to be free of psychiatric disorders, with no history of psychiatric/psychotherapeutic treatment or psychotropic medication use. In addition, HC were required to score ≤ 3 on the ASRS v1.1 screener [30] and ≤ 19 on the WURS-k [31].

### Assessment of demographics, medical history and psychometric testing

Participants completed questionnaires on demographic variables. Lifetime psychiatric disorders were assessed using the Mini-DIPS (Margraf & Cwik, 2017). A semi-structured interview obtained medical history, current medication, and smoking behavior (never vs. ever, current use). Verbal intelligence was estimated with the MWT-B [32]. ADHD symptoms were measured using the CAARS-S: L [33], and depressive symptoms with the QIDS-SR16 [34]. Physical activity, eating behavior, and sleep quality were assessed as cardiovascular risk-related factors [35], using the WHO Global Physical Activity questionnaire [36], the Intuitive Eating Scale IES-2 [37], the Epworth Sleepiness Scale [38], and the Pittsburgh Sleep Quality Index (PSQI) [39].

### Anthropometric measurements and 24-h BP recordings

Height was measured to the nearest centimeter using a Seca 206 rolling measuring tape, with participants wearing socks but no shoes. Weight was measured in underwear to the nearest kilogram with a calibrated Seca 877 scale. Body mass index (BMI) was calculated as weight (kg) divided by the squared height (m). Waist and hip circumference were measured to the nearest centimeter with a tape measure. Ambulatory 24-hour BP was recorded using custo screen 300 devices (custo med GmbH, Germany) at 15-minute intervals during daytime (06:00–22:00) and 30-min intervals at night. Systolic, diastolic and pulse values were exported by custo med software.

### Biomarkers

Blood was drawn by puncture of a cubital vein after participants had abstained from eating for at least 3 h. Serum samples were directly centrifuged for 5 min at 4500 g and supernatant frozen at − 80° C until further processed. All biomarkers (total cholesterol, HDL, LDL, triglycerides, glucose, interleukin 6 (IL-6), C-reactive protein (CRP)) were analyzed in the central laboratory unit of the University Hospital of Würzburg, Germany.

### Primary and secondary measures

The primary measure was the prevalence of MetS. We hypothesized that a higher proportion of adult ADHD patients would meet MetS criteria. MetS was defined using the Joint Interim Statement (JIS) harmonized IDF criteria [40], requiring at least three of the following risk factors: central obesity (waist circumference ≥ 94 cm in men, ≥ 80 cm in women), hypertension (systolic ≥ 130 mmHg and/or diastolic ≥ 85 mmHg), elevated triglycerides (≥ 150 mg/dl), reduced HDL cholesterol (< 40 mg/dl in men, < 50 mg/dl in women), and elevated fasting blood glucose (≥ 100 mg/dl). For glucose, we deviated from JIS by not requiring overnight fasting; participants abstained from eating for at least three hours. For hypertension, we used mean daytime systolic and diastolic values.

As secondary measures, we assessed MetS prevalence using IDF criteria [41], and NCEP ATP III criteria [42], which differ slightly from JIS and yield lower prevalence numbers [43, 44]. We also examined MetS components dimensionally, anthropometric measurements (height, weight, BMI, hip circumference, waist-to-hip ratio), verbal IQ, psychopathological, and behavioral variables, and smoking status. Finally, we analyzed inflammatory biomarkers interleukin-6 (IL-6) and C-reactive protein (CRP), LDL and total cholesterol. As measure of cardiovascular regulation, we analyzed 24-hour BP and HR profiles.

### Sample size calculation

A meta-analysis reported a pooled MetS prevalence of 4.8–7.0% in young adults (18–30 years) [45]. In contrast, a study of apparently healthy middle-aged Europeans (45–64 years) found a prevalence of 28.0% using IDF criteria, dropping to 8.9% in normal-weight individuals [46]. As our study focused on young to middle-aged adults without known hypertension, we expected MetS prevalence in HC to be low, around 10%. Evidence on MetS prevalence in adults with ADHD is limited. A small study of young adults (mean age 25 years) reported 10.8% [29], but the cohort was younger than ours. ADHD is associated with elevated obesity risk (28.2%, ~ 70% higher than the general population) [47], and roughly doubled odds for type 2 diabetes [18, 20]. In European middle-aged cohorts with major depression MetS prevalence ranges from 27% to 41% [48–50].

Based on these data, we expected an elevated MetS prevalence of ~ 30% in ADHD. Assuming MetS proportions of 10% in HC and 30% in ADHD, the required sample size per group for 90% power at α = 0.05 was *n* = 74 using one-sided Fisher’s exact test (G*Power v3.1.9.7). To account for missing data, we recruited a larger cohort. Of 170 initial participants, three (1 ADHD, 2 HC) were excluded due to lipid-lowering medication, and two patients for missing data, yielding a final sample of 165 (83 ADHD, 82 HC).

### Statistical analyses

All analyses were performed in SPSS v29. Differences in MetS prevalence between ADHD and HC were assessed using one-sided Fisher’s exact test, based on the a priori hypothesis of higher prevalence in ADHD. Between-group differences in categorical and continuous variables, including demographics, clinical variables, MetS components, anthropometric measurements, biomarkers, and BP and HR, were analyzed with χ² tests and two-sided Student’s t tests, respectively. CRP and IL-6 values below the assay’s detection limit were replaced with half that limit. 24-h BP and HR patterns were examined using two-way repeated-measures ANOVA with diagnosis as the between-subject factor and time (24 hourly means) as the within-subject factor, with Greenhouse-Geisser correction due to violation of the sphericity assumption. Hourly between-group comparisons used Bonferroni-corrected t tests across 24 time points. Significance was set at *p* < 0.05. For cardiovascular measures showing group differences, we further examined the effect of psychotropic medication (any medication; paused psychostimulant medication only; or non-stimulant medication vs. no medication) and comorbidity (ever vs. never) within the ADHD group.

Bivariate correlations explored associations among age, verbal IQ, psychopathological, behavioral, and metabolic variables. Based on 24-h BP findings, we tested a serial mediation model linking ADHD diagnosis (X) to nighttime systolic BP (Y), with HI (M1), eating behavior (M2), and BMI (M3) as mediators hypothesizing that impulsivity contributes to dysregulated eating [51], thereby increasing BMI, which is associated with higher BP [52]. Equivalent models were tested using inattention and depressive symptoms as M1.

Mediation analyses used bias-corrected bootstrapping (5,000 resamples) to estimate total, direct, and indirect effects, focusing on the serial path ADHD → HI → eating behavior → BMI → nighttime systolic BP. Primary covariates were age and sex; sensitivity analyses additionally included psychotropic medication, lifetime smoking status, depressive symptoms, and sleep quality. Physical activity and verbal IQ were excluded, as they were not associated with X or Y. Analyses were conducted using PROCESS macro (model 6) for SPSS (Hayes & Rockwood, 2017), with significance defined as a bootstrap 95% CI not crossing zero.

## Results

### Sample characteristics

The age distribution of the cohort is shown in Supplementary Fig. S1. Significantly more participants in the ADHD group reported having repeated a school year (31% vs. 12% in HC) or discontinued training or studies (40% vs. 13%; Table 1). There were no between-group differences in any other demographic variables (Table 1).

**Table 1:** Demographic variables

Variable	ADHD (*n* = 83)	HC (*n* = 82)	Statistics	Cohen’s D/OR [95% CI]	*p* value
*Sex*					
Female	*n* = 41 (49.4%)	*n* = 39 (47.6%)	χ^2^ = 0.1	0.96 [0.70, 1.32]	0.813
Male	*n* = 42 (50.6%)	*n* = 43 (52.4%)			
Age	M = 33.75 (11.26)	M = 34.89 (12.79)	t = 0.6	− 0.10 [− 0.40; 0.21]	0.543
*Place of residence*					
< 10,000 inhabitants	*n* = 26 (31.3%)	*n* = 18 (22.2%)	χ^2^ = 5.1	NA	0.079
10,000–100,000	*n* = 11 (13.3%)	*n* = 5 (6.2%)			
> 100,000	*n* = 46 (55.4%)	*n* = 58 (71.6%)			
NA	–	*n* = 1			
*Marital status*					
Single	*n* = 59 (71.1%)	*n* = 51 (62.2%)	χ^2^ = 4.9	NA	0.302
Married	*n* = 19 (22.9%)	*n* = 29 (35.4%)			
Divorced	*n* = 3 (3.6%)	*n* = 2 (2.4%)			
Living separately	*n* = 1 (1.1%)	–			
Widowed	*n* = 1 (1.1%)	–			
*Number of children*					
0	*n* = 60 (72.3%)	*n* = 56 (68.3%)	χ^2^ = 4.1	NA	0.256
1	*n* = 9 (10.8%)	*n* = 4 (4.9%)			
02-Mar	*n* = 12 (14.5%)	*n* = 20 (24.4%)			
> 3	*n* = 2 (2.4%)	*n* = 2 (2.4%)			
*Number of siblings*					
0	*n* = 11 (13.3%)	*n* = 8 (9.8%)	χ^2^ = 1.3	NA	0.737
1	*n* = 36 (43.4%)	*n* = 35 (42.7%)			
02-Mar	*n* = 30 (36.1%)	*n* = 35 (42.7%)			
> 3	*n* = 6 (7.2%)	*n* = 4 (4.9%)			
*Living situation*					
Alone	*n* = 21 (25.3%)	*n* = 15 (18.3%)	χ^2^ = 8.7	NA	0.193
With partner	*n* = 19 (22.9%)	*n* = 21 (25.6%)			
With partner and child(ren)	*n* = 15 (18.1%)	*n* = 19 (23.2%)			
Alone with child(ren)	*n* = 8 (9.6%)	*n* = 4 (4.9%)			
With partner	*n* = 14 (16.9%)	*n* = 22 (26.8%)			
With others	*n* = 3 (3.6%)	*n* = 1 (1.2%)			
Other	*n* = 3 (3.6%)	–			
*Graduation*					
Lower secondary education	*n* = 12 (14.5%)	*n* = 4 (4.9%)	χ^2^ = 4.4	NA	0.11
Secondary education	*n* = 17 (20.5)	*n* = 17 (20.7%)			
High school diploma	*n* = 54 (65.1%)	*n* = 61 (74.4%)			
Classes repeated	*n* = 26 (31.3%)	*n* = 10 (12.2%)	χ^2^ = 8.9	3.28 [1.46; 7.37]	**0.003**
Discontinued training/studies	*n* = 33 (39.8%)	*n* = 11 (13.4%)	χ^2^ = 14.6	4.26 [1.97; 9.22]	**< 0.001**
*Net household income*					
< 800 €/month	*n* = 2 (2.6%)	*n* = 7 (9.5%)	χ^2^ = 11.1	NA	0.05
801–1500 €/month	*n* = 16 (20.8%)	*n* = 7 (9.5%)			
1501–2000 €/month	*n* = 9 (11.7%)	*n* = 6 (8.1%)			
2001–3000 €/month	*n* = 22 (28.6%)	*n* = 14 (18.9%)			
3001–5000 €/month	*n* = 18 (23.4%)	*n* =23 (31.1%)			
> 5000 €/month	*n* = 10 (13.0%)	*n* = 17 (23.0%)			
NA	*n* = 6	*n* = 8			
Household size	M = 2.43	M = 2.46	t_160_ = 0.1	− 0.02 [− 0.33; 0.29]	0.891
*Employment status*					
Full-time	*n* = 28 (23.7%)	*n* = 37 (46.3%)	χ^2^ = 8.9	NA	0.114
Part-time	*n* = 14 (16.9%)	*n* = 10 (12.5%)			
In training/studying	*n* = 32 (38.6%)	*n* = 31 (38.8%)			
Part-time due to sickness	*n* = 3 (3.6%)	–			
Unemployed	*n* = 2 (2.4%)	*n* = 2 (2.5%)			
Unemployed due to sickness	*n* = 4 (4.8%)	–			
NA	–	*n* = 2			

*ADHD* attention-deficit/hyperactivity disorder, *HC* healthy controls, *OR* odds ratio, *CI* confidence interval, *NA* not available

Lifetime psychiatric comorbidity was common in the ADHD cohort: 64 (77.1%) patients had at least one comorbid disorder, according to Mini-DIPS interviews (Supplementary Fig. S2). Most frequent were affective disorders (54% unipolar, 6% bipolar, 5% dysthymia), followed by anxiety disorders (16% social phobia, 13% specific phobia, 6% generalized anxiety disorder, 6% agoraphobia, 5% panic disorder), substance use disorders (11%), obsessive-compulsive disorder (8%), posttraumatic stress disorder (7%), and eating disorders (5%) (Supplementary Fig. S2). Fifteen participants (18.1%) were taking antidepressants, 33 (39.8%) psychostimulants (paused), and three medical cannabis (paused); one used atomoxetine and two quetiapine. No significant differences in somatic comorbidity were observed between ADHD and HC (Supplementary Table S1).

ADHD and HC did not differ in verbal IQ. Regarding psychopathological and behavioral variables patients reported higher ADHD and depressive symptoms, poorer sleep, and less intuitive eating. They also showed later bedtimes and higher rates of current and lifetime smoking (Supplementary Table S2).

### MetS prevalence and MetS components

To address the main study objective, we first examined whether MetS prevalence differed between ADHD patients and healthy controls. Overall, 30 participants (18.2%) met JIS criteria for MetS in the full cohort. Mean age did not differ between participants with and without MetS (MetS: M = 36.7, SD = 13.0, no MetS: M = 33.8, SD = 11.8, t = − 1.22, *p* = 0.224). Within the ADHD group, 20 participants met MetS criteria (mean age = 36.3 years, median = 32.5 years; 10 female, 10 male), and within the HC group, 10 participants met criteria (mean age = 37.7 years, median = 36 years; 6 female, 4 male). Fisher’s exact test showed a significantly higher proportion of MetS in the ADHD group (OR = 2.29, *p* = 0.037), Table 2. With IDF criteria [41] or NCEP ATP III criteria [42] the group difference was not significant (IDF: OR = 2.14, *p* = 0.111; NCEP ATP III: OR = 2.01, *p* = 0.099) due to lower overall MetS numbers (Supplementary Table S3). For sex differences regarding MetS and its components, see Supplementary Table S4. Among MetS components, only the categorical waist circumference criterion was associated with ADHD status (Table 2). No group differences were observed for the other MetS components, in either categorical analyses or dimensional measures (Table 2; Supplementary Table S5).

ADHD patients with non-stimulant medication only (*n* = 12) were more likely to meet MetS criteria (*n* = 7, 58.3%) than those without psychotropic medication (6 of 38, 15.8%; χ² = 8.58, OR = 7.47 [1.77; 31.56], *p* = 0.003), whereas psychostimulant-only patients (*n* = 26; paused during assessments) were not (*n* = 5, 19.2%, *p* = 0.720). Patients with MetS also had more lifetime psychiatric comorbidities (2.05 vs. 1.40, *p* = 0.044, one-sided t-test), and a higher number of comorbidities corresponded to higher MetS prevalence (Supplementary Fig. S3). Prescription of psychotropic drugs (all, psychostimulants only, others only) was not associated with waist circumference or BMI in the ADHD group (all *p* > 0.05). Psychiatric comorbidity was linked to lower HDL, t(81) = 2.96, *p* = 0.004, and a trend towards higher odds of being in the HDL risk group (OR = 7.02 [0.87; 56.57], p = 0.038 χ² test, p = 0.059 Fisher's exact test). The effect of smoking on MetS and its components is reported in Supplementary Material.

**Table 2:** Proportions and statistics for MetS and MetS components in ADHD vs. HC

Variable	ADHD	HC	χ^2^	OR (95% CI)	*p* value
*MetS*					
Yes	*n* = 20 (24.1%)	*n* = 10 (12.2%)	NA	2.29 (1.14; ∞)	**0.037**
No	*n* = 63 (75.9%)	*n* = 72 (87.8%)			
*Waist circumference ≥ 80 cm (♀) or ≥ 94 cm (♂)*					
Yes	*n* = 40 (48.2%)	*n* = 18 (22.0%)	12.5	3.31 (1.68; 6.51)	**< 0.001**
No	*n* = 43 (51.8%)	*n* = 64 (78.0%)			
*RR sys ≥ 130 and/or RR dia ≥ 85*					
Yes	*n* = 44 (53.0%)	*n* = 34 (42.5%)	1.8	1.53 (0.82; 2.83)	0.179
No	*n* = 39 (47.0%)	*n* = 46 (57.5%)			
NA	–	*n* = 2			
*Triglycerides ≥ 150 mg/dl*					
Yes	*n* = 21 (25.3%)	*n* = 20 (24.4%)	0	1.05 (0.52; 2.13)	0.892
No	*n* = 62 (74.7%)	*n* = 62 (75.6%)			
*HDL cholesterol < 50 (♀) or < 40 (♂) mg/dl*					
Yes	*n* = 20 (24.1%)	*n* = 19 (23.2%)	0	1.05 (0.51; 2.16)	0.889
No	*n* = 63 (75.9%)	*n* = 63 (76.8%)			
*Glucose ≥ 100 mg/dl*					
Yes	*n* = 11 (13.3%)	*n* = 8 (9.8%)	1.4	1.41 (0.54; 3.72)	0.482
No	*n* = 72 (86.7%)	*n* = 74 (90.2%)			

*ADHD* attention-deficit/hyperactivity disorder, *HC* healthy controls, *OR* odds ratio, *CI* confidence interval, *MetS* metabolic syndrome, *RR sys* systolic blood pressure, *RR dia* diastolic blood pressure, *NA* not available, significant *p* values (p < .05) in bold.

### Anthropometric measurements

**Fig. 1:**
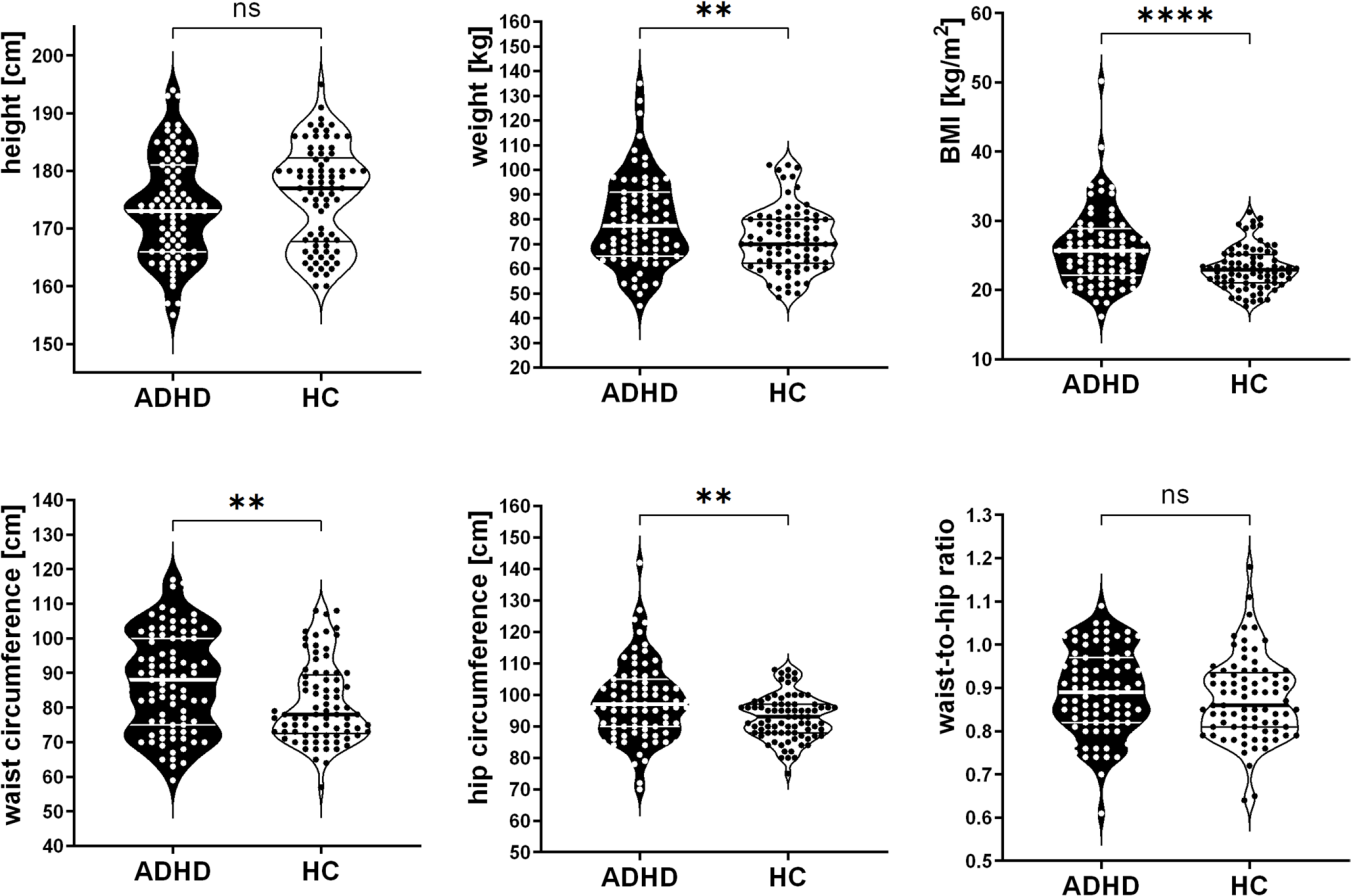
Group comparisons of anthropometric measurements. Violin plots (black: ADHD, white: HC) with individual measurement points. Weight, BMI, waist circumference, and hip circumference with significant differences; height and waist-to-hip ratio nonsignificant (ns). Level of significance of p values: *< 0.05, **< 0.01, ****< 0.0001, two-sided Student’s t test. *ADHD* attention-deficit/hyperactivity disorder, *HC* healthy controls, *BMI* body mass index

In addition to increased waist circumference (ADHD: M = 87.3, SD = 14.3 cm, HC: M = 81.3, SD = 11.6 cm, t(162) = − 2.97, *p* = 0.003), the ADHD group showed greater hip circumference (M = 97.9, SD = 12.3 cm) compared with the HC group (M = 92.7, SD = 7.2 cm), t(162) = − 3.29, *p* = 0.001. Patients also had higher body weight (M = 79.1, SD = 18.2 kg) than HC (M = 71.8, SD = 12.7 kg), t(163) = − 2.98, *p* = 0.003, as well as higher BMI (ADHD: M = 26.1, SD = 5.4; HC: M = 23.1, SD = 3.0), t(163) = − 4.38, *p* < 0.0001. There was a significant association between ADHD status and weight category (BMI < 18.5: underweight, BMI 18.5–24.9: normal weight, BMI 25–29.9: overweight, BMI ≥ 30: obese), χ^2^ (3, *n* = 165) = 20.63, *p* < 0.001, with 37% of patients overweight (vs. 23% of HC), and 19% obese (vs. 2% in HC), as shown in Supplementary Fig. S4. No significant group differences were found for body height or waist-to-hip ratio (WHR; Fig. 1).

### Blood lipids and inflammatory markers

In addition to nonsignificant results for triglycerides and HDL, no group differences were observed for total cholesterol (ADHD: M = 182.7, SD = 40.0 mg/dl; HC: M = 190.6, SD = 40.3 mg/dl, t_163_ = 1.28, *p* = 0.204), or LDL (ADHD: M = 108.3, SD = 32.9 mg/dl; HC: M = 117.0, SD = 32.6 mg/dl, t_163_ = 1.69, *p* = 0.093). Similarly, no evidence of increased inflammation in the ADHD group was found, as indicated by nonsignificant findings for CRP (ADHD: M = 0.16, SD = 0.30 mg/dl; HC: M = 0.14, SD = 0.16 mg/dl, t_163_ = −0.42, *p* = 0.675), and IL-6 (ADHD: M = 1.62, SD = 1.63 mg/dl; HC: M = 1.73, SD = 1.44 mg/dl, t_163_ = 0.46, *p* = 0.647), Supplementary Table S6.

### 24-hour BP and HR measurements

**Fig. 2:**
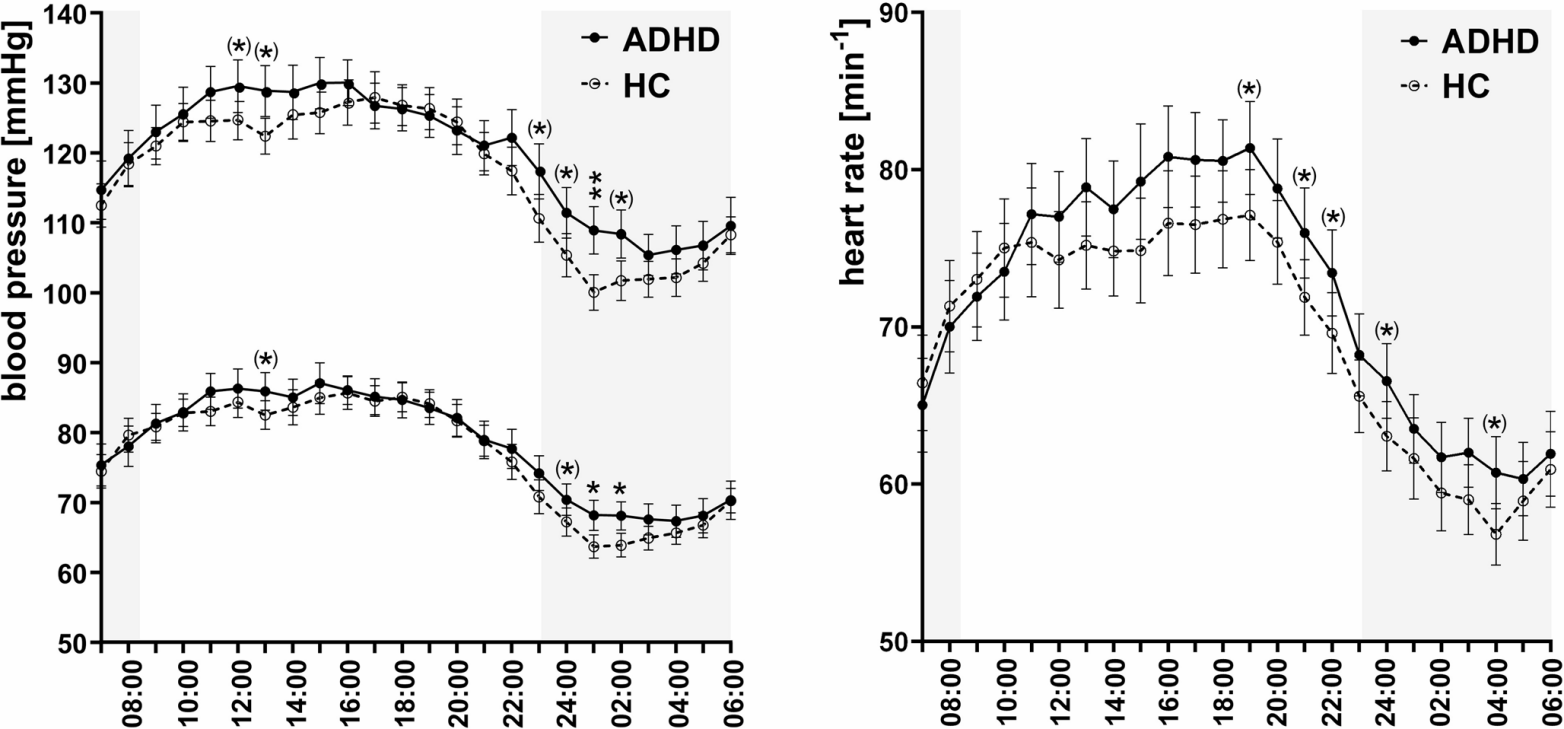
24-hour blood pressure and heart rate profiles for ADHD and HC participants. Left Hourly mean systolic (above) and diastolic (below) values. Right Hourly mean heart rates. Error bars indicate 95% confidence intervals. Solid line: ADHD, dashed line: HC. Significance levels: *< 0.05, **< 0.01 (Bonferroni-corrected for 24 time points), (*) < 0.05 (nominally significant values not corrected for multiple testing). Grey background indicates the mean nighttime for the full cohort, transparent background mean daytime. *ADHD* attention-deficit/hyperactivity disorder, *HC* healthy controls

A repeated-measures ANOVA revealed a significant time × diagnosis interaction for systolic blood pressure, F(11.32, 491.0) = 2.31, *p* = 0.008, η² = 0.02, indicating that the 24-hour systolic profile differed between groups, whereas this interaction was not significant for 24-hour diastolic BP, F(10.33, 198.14) = 1.52, *p* = 0.125, and HR measures, F (9.92, 265.99) = 1.34, *p* = 0.202. Patients showed a higher nighttime systolic mean (ADHD M = 108.1 (13.9) mmHg; HC M = 103.8 (10.3) mmHg, t_161_ = 2.21, *p* = 0.029), and a higher 24-h HR mean (ADHD M = 72.0 (8.9) min^− 1^, HC M = 69.3 (8.4) min^− 1^, t_161_ = 1.98, *p* < 0.05). Within the ADHD group, neither lifetime psychiatric comorbidity (never vs. ever) nor psychotropic medication (all, psychostimulants only, others only vs. no medication) was associated with 24-h HR or nighttime systolic BP (all two-sided t tests > 0.05). BP and HR were nominally elevated throughout most of the day in patients. After Bonferroni correction (24 time points), mean systolic and diastolic means remained significantly higher in the ADHD group at 1 a.m. (and 2 a.m. for mean diastolic values). Several additional time windows showed higher BP and HR in the ADHD group (*p* < 0.05), though these did not survive multiple comparison corrections (Fig. 2). Additionally, a higher proportion of diastolic non-dippers was observed in the ADHD group (ADHD: *n* = 9, 11%; HC: *n* = 2, 2.5%; χ² = 4.60, *p* = 0.032). However, diastolic non-dippers were overrepresented among psychostimulant users (*n* = 6, 23.1% vs. *n* = 2, 5.4%, χ² = 4.30, OR = 5.25 [0.97; 28.51], *p* = 0.038). Further details of 24-h recordings are provided in Supplementary Table S7.

### Mediation analyses

Bivariate correlations between psychopathological symptoms, behavioral factors, BMI, and verbal IQ are shown in Supplementary Fig. S5 and S6. Only BMI and HI were significantly associated with nighttime systolic BP. Disordered eating behavior, reflected by lower IES-2 scores, correlated strongly with BMI, and also with HI.

**Fig. 3:**
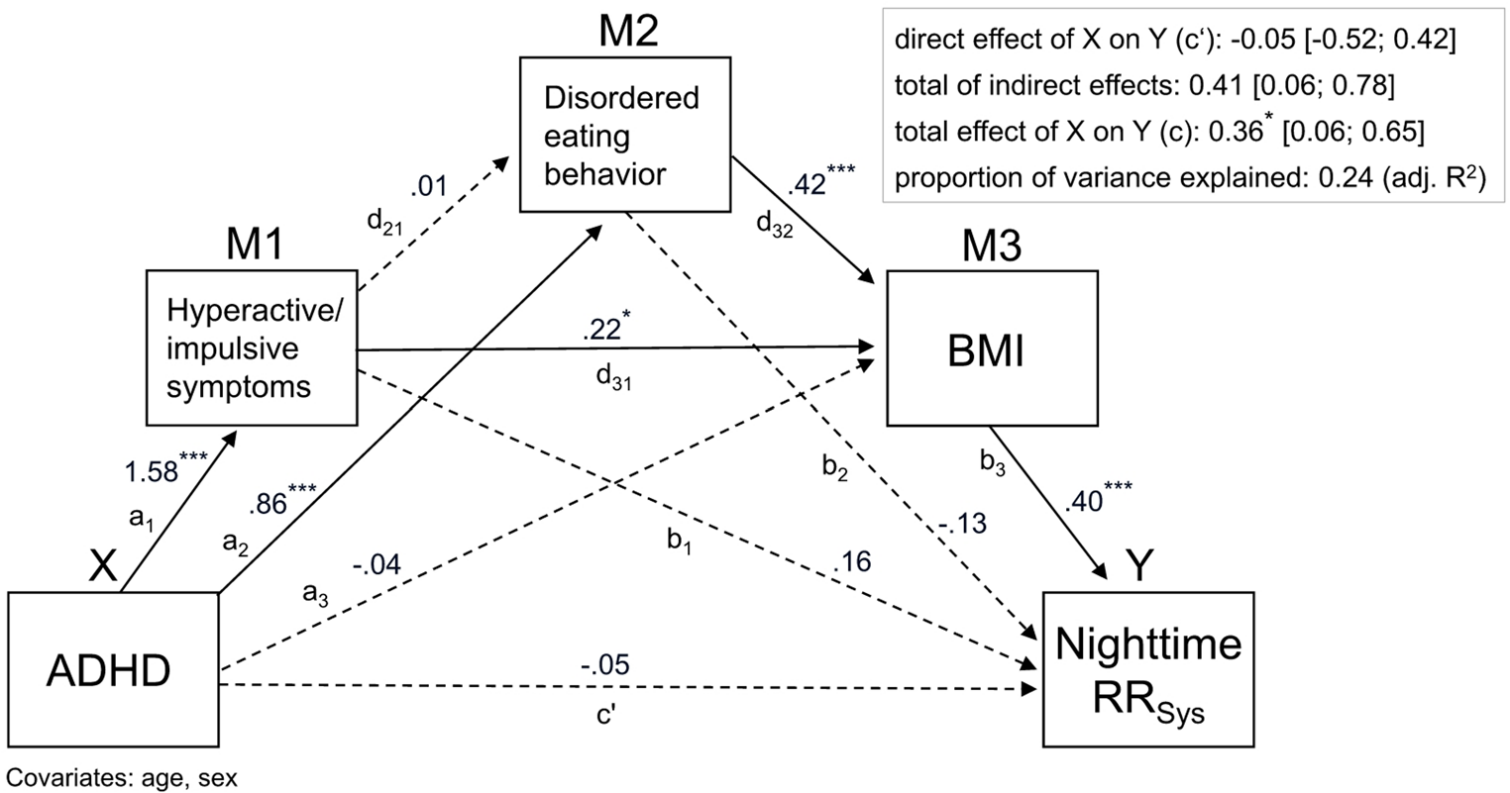
Serial mediation model. The association between ADHD and elevated nighttime systolic blood pressure is fully mediated by a mediation model with hyperactivity/impulsivity (M1), disordered eating behavior (M2), and body mass index (BMI, M3) as serial mediators. Solid arrows show significant paths, dashed arrows show nonsignificant paths. Individual paths are labeled with their standardized beta weights. Covariates: Age, sex. *< 0.05, **< 0.01, ***< 0.001. *ADHD* attention-deficit/hyperactivity disorder, *RR*_*Sys*_ systolic blood pressure, *BMI* body mass index, disordered eating behavior assessed by IES-2 inverted total scores

Consequently, we fitted a mediation model to examine the association between ADHD (X) and nighttime systolic BP (Y), with HI (M1), disordered eating (M2), and BMI (M3) as serial mediators, adjusting for age and sex. The total effect (c) of diagnosis on nighttime systolic BP remained significant in this model. When the mediators were included, the direct effect (c′) was no longer significant, whereas the total indirect effect was significant, indicating full mediation (Fig. 3), although the longest serial path from X to Y via M1, M2, and M3 was not significant. The model explained 24% of the variance (Fig. 3). Sensitivity analyses including additional covariates confirmed the significance of the total indirect effects, again suggesting full mediation (Fig. S7 A).

The model with inattention as M1 was nonsignificant when adjusted for age and sex (Supplementary Fig. S7B), but became significant after including additional covariates. In this model, the serial path from X to Y via M1, M2, and M3 was significant (Supplementary Fig. S7C). Alternative mediation models using depressive symptoms as M1 were not significant (Supplementary Fig. S7D and E).

## Discussion

In this study, apparently healthy young to middle-aged adults with ADHD had twice the odds of MetS compared to HC (24% vs. 12%). They also showed elevated nighttime systolic BP, higher 24-h HR, and a greater likelihood of diastolic non-dipping. Nighttime systolic elevation was mediated by HI, disordered eating, and BMI. These findings suggest a pattern consistent with early cardiovascular risk in adults with ADHD and point to possible pathways from ADHD symptoms to dysregulated eating, obesity, and BP. MetS was more common in ADHD patients with multiple psychiatric comorbidities and in those taking non-stimulant psychiatric medication such as antidepressants. Additionally, in the patient group there was a significantly higher proportion of past and current smokers than in HC, in line with the increased prevalence of smoking and other addictive behaviors in ADHD [53]. As a major cardiovascular risk factor [54, 55], smoking was associated with a higher likelihood of meeting MetS criteria. Thus, the association between ADHD and MetS may partly reflect the influence of psychiatric comorbidity, smoking behavior, and/or non-stimulant medication, rather than factors inherent to ADHD alone.

Because we recruited participants without known cardiovascular disease and without cardiac or lipid-lowering medication, comparison of our MetS rates with population-based studies is limited. Overweight and obesity were lower in our HC group (26%) than in the German GEDA study (54%) [56], indicating that we recruited a particularly healthy sample in line with a similar prevalence in an apparently healthy European cohort [46]. MetS prevalence varies with diagnostic criteria. As our main interest was in early cardiovascular risk in apparently healthy adults, we intentionally chose the more sensitive JIS criteria, which typically produce higher estimates than IDF or ATP III definitions [44]. Applying these less sensitive definitions did not yield significant group differences in our cohort, although odds ratios remained around 2. A previous study in 158 adult outpatients with an ADHD diagnosis reported a MetS prevalence of 10.8%, using ATP III criteria [29]. However, this study had no comparable group of HC or psychiatric outpatients without an ADHD diagnosis to enable an attribution of MetS specifically to ADHD. In our ADHD cohort, MetS prevalence was somewhat higher than in the study of di Girolamo et al. [29]. This difference can possibly be explained by the higher mean age in our cohort (34 years vs. 25 years). For early cardiovascular screening, the greater sensitivity of the JIS definition may be advantageous [57]. Given that MetS predicts myocardial infarction, stroke, and cardiovascular mortality [58], higher MetS rates in ADHD may indicate elevated cardiovascular risk in addition to the well-established excess mortality from accidents [8, 9], paralleling risks observed in severe psychiatric disorders such as major depression and schizophrenia [59].

Waist circumference was the only MetS component elevated in ADHD. Its large effect suggests that the association between ADHD and MetS is primarily driven by obesity, a well-known comorbidity of ADHD [17, 47]. Total cholesterol, HDL, LDL, and triglycerides did not differ between ADHD and HC. Although some studies report altered lipid profiles in children with ADHD [60–63], findings are inconsistent, with both lower and higher LDL levels described. Psychiatric comorbidity was associated with lower HDL, consistent with findings in major depression [64]. Overall, current evidence, including our results, does not support a generally altered lipid metabolism in ADHD. In addition to the absence of differences in blood lipids, there was also no systematic difference between ADHD patients and HC regarding serum glucose levels in our cohort. Thus, while individuals meeting MetS criteria necessarily had at least three metabolic risk factors, the group difference in MetS prevalence appears to be largely influenced by higher rates of central obesity in the ADHD group, rather than by systematic group differences across the full range of metabolic abnormalities. There is evidence from a large prospective study that central obesity precedes the development of other MetS components [65]. Hence, the cardiometabolic profile found in our cohort may be indicative of an early metabolic risk pattern. Notably, in contrast to MetS, increased waist circumference was not associated with psychiatric comorbidity or psychotropic medication, suggesting that the association with obesity might rather be related to behavioral and/or neurobiological correlates of ADHD.

Inflammatory markers were not elevated in adults with ADHD. Inflammation has been proposed as a shared mechanism linking psychiatric and cardiovascular disorders [66], and is associated with obesity [67]. CRP and IL-6 did not differ in our ADHD cohort. Prior studies on inflammation in ADHD showed mixed results [68]. It is possible that inflammatory changes occur only in a subgroup of patients [69] or during acute episodes of comorbid psychiatric disorders, which were not present in our sample at the time of assessment.

The ADHD group showed signs of altered cardiovascular regulation. In particular, there was an altered circadian pattern of systolic BP in ADHD. Nighttime systolic BP (increase of 4 mmHg) and HR (increase of 3 bpm) were slightly higher. In addition, there was an increased likelihood of diastolic non-dipping, potentially linked to psychostimulant use. Patients paused stimulant medication one week prior to study investigations. However, longer-term stimulant effects on blood pressure cannot be fully ruled out [70]. Our findings indicate subtle alterations in BP and HR regulation. They may reflect heightened autonomic arousal, consistent with sympathetic-parasympathetic dysregulation [71, 72] and reduced vagally mediated heart-rate variability in ADHD [72]. Thus, cardiovascular risk in ADHD may not only be due to obesity, but might also stem from alterations in autonomic arousal and diurnal cardiovascular regulation.

Elevated nighttime systolic BP in ADHD appears to arise from symptom-related behavioral and metabolic pathways. We focused on nighttime systolic BP because it more strongly predicts cardiovascular events and outcomes than daytime values [73, 74]. We found that higher nighttime systolic BP in ADHD was fully mediated by HI, disordered eating, and BMI, although the full serial pathway was not significant because HI did not predict eating behavior in this model. Thus, elevated nighttime systolic BP in ADHD appears to be mediated by disordered eating and overweight, with BMI influenced directly by HI. Prior work supports links between impulsivity and disordered eating [75, 76]. The correlation between BMI and BP is also well-known [77, 78]. Our data support the notion that behavioral eating-related factors and weight-related factors may shape cardiovascular risk in ADHD unfavorably. BMI was directly influenced by HI in our cohort. Altered mesolimbic dopamine signaling may contribute to both dysregulated food intake and energy expenditure (Dunigan & Roseberry, 2022; Timper & Bruning, 2017). Hence, besides impulsivity-driven overeating, a broader dysfunction in reward-related brain networks may jointly affect ADHD symptoms and basal metabolic rates, reflective of a direct pathway from HI to increased BMI.

Our study has several strengths and limitations. To our knowledge, it is the first to compare MetS prevalence in adult ADHD using a case-control design. Beyond replicating the established association of ADHD with obesity, it provides new insights into diurnal HR and BP regulation and highlights possible psychopathological and behavioral pathways linking ADHD to higher nighttime BP. However, the sample size was rather small, and the cross-sectional design limits causal inference, underscoring the need for replication in larger and longitudinal cohorts. Another limitation arises from the high lifetime comorbidity of ADHD with other psychiatric disorders (77% of patients in our cohort), which makes it difficult to ascribe group differences specifically to ADHD when comparing to healthy controls. Future studies could make use of a psychiatric control group without ADHD, in addition to HC, to separate ADHD-specific from more global effects of psychiatric disorders. In our ADHD cohort, a higher number of comorbid psychiatric disorders and non-stimulant psychotropic medication was associated with MetS, possibly hinting at effects from other psychiatric disorders. Such effects might be further augmented by smoking, which is generally common in psychiatric disorders [79], and adds to other detrimental behavioral variables, such as low physical activity and unhealthy diet [25]. Several clinical variables (e.g. eating behavior, sleep, and physical activity) were assessed solely through self-report, which may have biased results. Notably, self-reported physical activity did not differ between groups, contrary to expectations of lower activity in ADHD [80], leading us to exclude it as a covariate. Future studies should incorporate objective methods such as actigraphy, sleep tracking, or momentary assessments of food intake.

In conclusion, physically healthy young to middle-aged adults with ADHD in this cohort showed a higher MetS prevalence when JIS criteria were applied. This effect was largely driven by differences in obesity and potentially influenced by psychiatric comorbidity and non-stimulant psychotropic medication. This finding in a cohort that was intentionally recruited to comprise apparently healthy participants without a known history of cardiovascular disease supports an early cardiovascular-risk trajectory in ADHD, in line with previous evidence [23]. This risk appears to be shaped both by psychiatric comorbidity and psychotropic medication, and by ADHD-related overeating, overweight, and autonomic overarousal. Thus, adults with ADHD face not only a substantial psychiatric burden but may also be at increased cardiovascular risk. Hence, early cardiovascular screening approaches and weight-management interventions should be considered in adult patients with ADHD.

## Supplementary Information

Below is the link to the electronic supplementary material.

Supplementary file1

## Data Availability

The datasets generated and analyzed during the current study are available from the corresponding author upon reasonable request.
