# Supplementary material for: ADHD and metabolic syndrome: behavioral and weight-related pathways to cardiovascular risk
Source: Eur Arch Psychiatry Clin Neurosci. 2026 May 19;276(5):2411–23. doi: 10.1007/s00406-026-02246-6 (PMC13481769; doi:10.1007/s00406-026-02246-6)
Supplement: Supplementary file 1 [file 406_2026_2246_MOESM1_ESM.docx]

**Supplementary Material**

**ADHD and Metabolic Syndrome: Behavioral and Weight-Related Pathways to Cardiovascular Risk**

Georg C. Ziegler, Katharina Kürten, Karla Roshop, Matthias Nieberler, Bodo Warrings, Sarah Kittel-Schneider, Klaus-Peter Lesch, Martin J. Herrmann, Stefan Störk, Jürgen Deckert

^1^ Department of Psychiatry, Psychosomatics and Psychotherapy, University Hospital of Würzburg, Würzburg, Germany

^2^ Department of Psychiatry and Neurobehavioural Science, University College Cork, Ireland

^3^ Department of Child- and Adolescent Psychiatry, Psychosomatics and Psychotherapy, Center of Mental Health, University of

Würzburg, Germany

^4^ Department of Psychiatry and Neuropsychology, School for Mental Health and Neuroscience (MHeNs), Maastricht University,

The Netherlands

^5^ Comprehensive Heart Failure Center (CHFC), University Clinic Würzburg, Germany

^6^ Department of Internal Medicine I, University Hospital Würzburg, Germany

^7^ Institute of Clinical Epidemiology and Biometry, University of Würzburg, Germany

**The effect of smoking status in MetS and MetS components in ADHD and HC**

In the full cohort all-time smokers were more likely to have MetS (27.4%) than never-smokers (13.1%), χ^2^ (n = 161) = 5.13, OR = 2.50 [1.12; 5.60] p < .023. This effect was driven by the ADHD group χ^2^ (n = 81) = 4.58, OR = 3.31 [1.07; 10.25] p < .032, whereas the effect of smoking on MetS was not visible in HC (p > .05); however, these differences did not amount to a significant smoking x ADHD interaction term in a logistic regression model (OR = 3.92 [0.53; 28.91]). On the level of MetS components smoking was associated with the waist circumference (χ^2^ = 7.58, OR = 2.54 [1.30; 4.99], p = .006) and the blood pressure criterion (χ^2^ = 4.29, OR = 1.97 [1.03; 3.80], p = .038). For blood pressure, the smoking x ADHD interaction term was significant in a logistic regression model (OR = 6.47 [1.53; 27.36], p = .011), as all-time smokers were more likely in the high blood pressure group within the ADHD group (68.2% vs. 35.1%, χ^2^ = 8.81, OR = 3.96 [1.57; 9.99], p = .003) but not in HC (OR = 0.61 [0.20; 1.84]). Smoking status was not associated with other MetS components (serum glucose, HDL, LDL, all p values of two-sided Chi-square tests > .05, data not shown).

**Supplementary Figures**

**Fig. S1** Age distribution of the ADHD and HC cohort. The x-axis represents age in years (in 3-year steps), the y-axis number of participants. ADHD: Attention-deficit/hyperactivity disorder, HC: Healthy controls

**Fig. S2** Psychiatric comorbidity in the ADHD group. The left graph represents the percent distribution of cumulative numbers of comorbid psychiatric disorders in the ADHD group. The right graph represents percentages of lifetime psychiatric comorbidity with different psychiatric disorders according to clinical interview and Mini-DIPS results in the ADHD group. GAD: Generalized anxiety disorder, PTSD: Posttraumatic stress disorder, OCD: Obsessive-compulsive disorder, SUD: Substance use disorder, PD: Personality disorder

**Fig. S3** Proportion of MetS in ADHD patients grouped in categories for number of lifetime psychiatric comorbidities. Statistical significance assessed by Chi-square tests. MetS: Metabolic syndrome

**Fig. S4** Proportion of participants in different BMI categories. ADHD: Attention-deficit/hyperactivity disorder, HC: Healthy controls. BMI: Body-mass-index

**Fig. S5** Correlation analysis. Bivariate correlation coefficients between age, verbal IQ, psychopathological variables, behavioral variables, BMI and nighttime systolic blood pressure. Verbal IQ assessed by MWT-B, inattention and hyper-activity/impulsivity by respective DSM-IV based symptoms assessed by CAARS-S:L, depressive symptoms assessed by QIDS-SR16, sleep problems assessed by PSQI, disordered eating behavior by IES-2, and physical activity by GPAQ. * p < .05, ** p < .01, p < .001

**Fig. S6** Scatter plots with linear regression lines for significant correlations of Fig. S5.

Data points for the ADHD group are black dots, data points for the HC cohort are empty points.

ADHD: Attention-deficit/hyperactivity disorder, HC: Healthy controls

**B**

**C**

**Dv**

**Ev**

**A**

**Fig. S7** Serial Mediation models. Different mediation models for the association between ADHD (X) and nighttime systolic blood pressure (Y) with disordered eating behavior as M2 and BMI as M3, and different mediator variables for M1 (A: Hyperactivity/impulsivity, B and C: Inattention, D and E: Depressive symptoms): Covariates: Age and sex (models B and D); Age, sex, psychiatric drug medication, smoking status, depressive symptoms (only in models where depressive symptoms is not M1), and sleep quality. Models A) and C) are significant for the total of indirect effects, indicating full mediation, whereas models B), D), and E) are not significant

**Supplementary Tables**

| **Variable** | **ADHD** | **HC** | **statistics** | **OR [95% CI]** | **p value** |
| --- | --- | --- | --- | --- | --- |
| Orthopedic | n = 11 (13.3%) | n = 10 (12.2%) | χ2 = 0.04 | 1.10 [0.44; 2.75] | .838 |
| Pulmonological | n = 7 (8.4%) | n = 5 (6.1%) | χ2 = 0.33 | 1.42 [0.43; 4.67] | .563 |
| Gastrointestinal | n = 8 (9.6%) | n = 3 (3.7%) | χ2 = 2.37 | 2.81 [0.72; 10.99] | .124 |
| Thyroid gland | n = 5 (6.0%) | n = 6 (7.3%) | χ2 = 0.11 | 0.81 [0.24, 2.77] | .739 |
| Dermatological | n = 6 (7.2%) | n = 1 (1.2%) | χ2 = 3.67 | 6.31 [0.74; 53.64] | .056 |
| Autoimmune | n = 6 (7.2%) | n = 1 (1.2%) | χ2 = 3.67 | 6.31 [0.74; 53,64] | .056 |
| Veinous | n = 5 (6.0%) | n = 1 (1.2%) | χ2 = 2.72 | 5.19 [0.59; 45.45] | .099 |
| Migraine | n = 2 (2.4%) | n = 3 (3.7%) | χ2 = 0.22 | 0.65 [0.11; 4.00] | .640 |
| Nephrological | n = 1 (1.2%) | n = 2 (2.4%) | χ2 = 0.35 | 0.49 [0.04; 5.49] | .553 |
| Oncological | n = 1 (1.2%) | n = 1 (1.2%) | χ2 = 0.00 | 0.99 [0.06,16.06] | .993 |
| Genetic | n = 0 (0.0%) | n = 2 (2.4%) | χ2 = 2.05 | 0.98 [0.94; 1.01] | .152 |
| Gynecological | n = 2 (2.4%) | n = 0 (0.0%) | χ2 = 2.00 | 1.03 [0.99; 1.06] | .157 |
| Blood | n = 1 (1.2%) | n = 1 (1.2%) | χ2 = 0.00 | 0.99 [0.06; 16.06] | .993 |
| Peripheral nerve | n = 2 (2.4%) | n = 0 (0.0%) | χ2 = 2.00 | 1.03 [0.99; 1.06] | .157 |
| Ophtalmological | n = 0 (0.0%) | n = 1 (1.2%) | χ2 = 1.02 | 0.99 [0.96; 1.01] | .313 |

**Table S1** Proportions and Chi-Square statistics for somatic disorder categories in ADHD and healthy controls. ADHD: Attention-deficit/hyperactivity disorder, HC: Healthy controls, OR: odds ratio, CI: confidence interval

| **Variable** | **ADHD** | **HC** | **statistics** | **Cohen’s D/OR [95% CI]** | **p value** |
| --- | --- | --- | --- | --- | --- |
| **Cognitive variable**  Verbal IQ (MWT-B) | M = 112.0 (14.5) | M = 115.4 (14.9) | t_162_ = 1.5 | -0.23 [-0.54; 0.08] | .145 |
| **Psychopathological variables**  ADHD symptom ratings  ASRS V1.1 (Screener)  WURS-K  CAARS IA/MP  CAARS HY/MR  CAARS I/EL  CAARS SCP  CAARS DSM IA  CAARS DSM HY/I  CAARS ADHD index | M = 4.6 (1.2)  M = 40.2 (11.9)  M = 73.8 (12.3)  M = 70.2 (11.7)  M = 65.5 (12.9)  M = 64.1 (14.3)  M = 75.7 (11.6)  M = 70.6 (12.9)  M = 73.8 (11.2) | M = 0.5 (0.8)  M = 7.0 (5.5)  M = 44.3 (7.9)  M = 45.7 (7.0)  M = 41.0 (7.1)  M = 45.6 (7.5)  M = 44.0 (6.7)  M = 43.9 (5.9)  M = 41.7 (6.4) | t_162_ = -25.6  t_162_ = -22.9  t_162_ = -18.3  t_162_ = -16.3  t_162_ = -15.1  t_162_ = -10.5  t_162_ = -21.6  t_162_ = -17.1  t_162_ = -22.6 | 4.00 [3.46; 4.53]  3.57 [3.07; 4.06]  2.85 [2.41; 3.28]  2.54 [2.12; 2.95]  2.34 [1.94; 2.74]  1.62 [1.27; 1.97]  3.35 [2.87; 3.82]  2.65 [2.23; 3.07]  3.50 [3.01, 3.99] | **< .001**  **< .001**  **< .001**  **< .001**  **< .001**  **< .001**  **< .001**  **< .001**  **< .001** |
| Depressive symptoms rating  QIDS-SR16 | M = 9.9 (4.9) | 2.5 (1.8) | t_163_ = -12.9 | 2.00 [1.62, 2.37] | **< .001** |
| **Behavioral variables**  Sleep  Epworth Sleepiness Scale  Average time to bed  Average time to get up  PSQI total score  PSQI sleep quality  PSQI sleep latency  PSQI sleep duration  PSQI sleep efficiency  PSQI sleep disturbances  PSQI sleeping medication  PSQI daytime dysfunction | M = 9.0 (4.1)  M = 23:14 (+/- 69 min)  M = 07:18 (+/- 92 min)  M = 9.2 (3.7)  M = 1.5 (0.7)  M = 1.6 (0.9)  M = 0.9 (1.0)  M = 0.8 (1.1)  M = 1.4 (0.6)  M = 1.5 (0.7)  M = 1.6 (0.7) | M = 6.3 (3.1)  M = 22:54 (+/- 52 min)  M = 07:00 (+/- 67 min)  M = 4.6 (2.)  M = 0.8 (0.6)  M = 0.7 (0.7)  M = 0.3 (0.6)  M = 0.3 (0.6)  M = 1.0 (0.3)  M = 0.8 (1.6)  M = 0.8 (0.7) | t_163_ = -4.7  t_161_ = -2.0  t_161_ = -1.4  t_163_ = -9.5  t_163_ = -6.9  t_163_ = -7.0  t_163_ = -4.8  t_163_ = -3.6  t_163_ = -5.7  t_163_ = -6.9  t_163_ = -7.8 | 0.73 [0.42; 1.05]  0.31 [0.00; 0.62]  0.22 [-0.09; 0.53]  1.48 [1.13; 1.83]  1.08 [0.75, 1.41]  1.08 [0.75; 1.41]  0.75 [0.43; 1.07]  0.57 [0.25; 0.88]  0.89 [0.56; 1.20]  1.08 [0.75, 1.41]  1.22 [0.88; 1.55] | **< .001**  **.047**  .158  **< .001**  **< .001**  **< .001**  **< .001**  **< .001**  **< .001**  **< .001**  **< .001** |
| Eating behavior  IES-2 total score  IES-2 UPE Scale  IES-2 EPRE Scale  IES-2 RHSC Scale  IES-2 BFCC Scale | M = 3.3 (0.6)  M = 3.5 (0.8)  M = 3.3 (0.9)  M = 3.2 (1.0)  M =3.1 (0.9) | M = 3.8 (0.5)  M = 3.7 (0.7)  M = 4.1 (0.7)  M = 3.8 (0.8)  M = 3.5 (0.8) | t_162_ = 6.2  t_162_ = 1.2  t_162_ = 6.7  t_162_ = 4.4  t_162_ = 4.0 | 0.97 [0.64; 1.29]  0.19 [-0.12; 0.50]  1.05 [0.72; 1.37]  0.69 [0.37; 1.00]  0.62 [0.31; 0.93] | **< .001**  .230  **< .001**  **< .001**  **< .001** |
| Physical activity  Moderate/vigorous per week  WHO recomm. fulfilled  NA | M = 296 (418) min  n = 43 (56.6%)  n = 7 | 365 (526) min  n = 56 (69.1%)  n = 1 | t_154_ = 0.9  χ2 = 2.7 | -0.14 [-0.46; 0.14]  0.58 [0.30; 1.12] | .371  .103 |
| Smoking behavior  Current smokers  All-time smokers  NA | n = 16 (19.3%)  n = 44 (54.3%)  n = 2 | n = 3 (3.7%)  n = 18 (22.5%)  n = 2 | χ2 = 9.9  χ2 = 17.2 | 6.29 [1.76; 22.73]  4.10 [2.07; 8.11] | **.002**  **< .001** |

**Table S2** Clinical characterization. ADHD: Attention-deficit/hyperactivity disorder, HC: Healthy control, M: mean, standard deviations (for dimensional variables) or percentage (for categorical variables) in brackets. N: sample size. T: t- statistics with d(f) as subscript. Significant (uncorrected) p values < .05 in bold. Verbal IQ: intelligence quotient as measured by a multiple-choice word test (MWT-B). ASRS V1.1: Adult ADHD Self-Report Scale. WURS-K: short version of the Wender-Utah Rating Scale. CAARS: self-report form of the long version of the Conners Adult ADHD Rating Scale, IA/MP: subscale assessing inattention and memory problems, HY/MR: subscale assessing hyperactivity and motor restlessness, I/EL: subscale assessing impulsivity and emotional lability, SCP: subscale assessing self-concept problems, DSM-IA: subscale assessing inattentive symptoms according to the DSM-IV, DSM-HY/I: subscale assessing hyperactive-impulsive symptoms according to the DSM-IV. PSQI: Pittsburgh Sleep Quality Index. IES-2: Intuitive Eating Scale 2 assessing four different subscale: Unconditional permission to eat (UPE), eating for physical rather than for emotional reasons (EPRE), reliance on hunger and satiety cues (RHSC), and body-food choice congruence (BFCC)

| **Variable** | **ADHD** | **HC** | **statistics** | **OR [95% CI]** | **p value** |
| --- | --- | --- | --- | --- | --- |
| MetS (IDF criteria)  yes  no | n = 12 (14.5%)  n = 71 (85.5%) | n = 6 (7.3%)  n = 76 (92.7%) | NA | 2.14 [0.90; ∞] | .111 |
| MetS (NCEP ATP-III criteria)  yes  no | n = 15 (18.1%)  n = 68 (81.9%) | n = 8 (9.9%)  n = 73 (69.6%) | NA | 2.01 [0.93; ∞] | .099 |

**Table S3** Proportions and statistics for MetS and MetS components in ADHD vs. HC with IDF and NCEP

ATP-III criteria. ADHD: Attention-deficit/hyperactivity disorder, HC: Healthy controls, OR: odds ratio, CI:

confidence interval, NA: not available, MetS: Metabolic syndrome, IDF: International Diabetes Federation, NCEP ATP-III: National Cholesterol Education Program Adult Treatment Panel III

| **Variable** | **Female** | **Male** | **statistics** | **OR [95% CI]** | **p value** |
| --- | --- | --- | --- | --- | --- |
| MetS (JIS criteria)  yes  no | n = 16 (20.0%)  n = 64 (80.0%) | n = 14 (16.5%)  n = 71 (83.5%) | χ2 = 0.35 | 1.27 [0.57; 2.80] | .557 |
| Waist circumference ≥ 80 cm (♀) or ≥ 94 cm (♂)  yes  no | n = 24 (30.4%)  n = 55 (69.6%) | n = 33 (38.8%)  n = 52 (61.2%) | χ2 = 1.29 | 0.69 [0.36; 1.32] | .257 |
| RR sys ≥ 130 and/or RR dia ≥ 85  yes  no  NA | n = 31 (39.7%)  n = 47 (60.3%) | n = 47 (55.3%)  n = 38 (44.7%) | χ2 = 3.94 | 0.53 [0.29; 1.00] | **.047** |
| Triglycerides ≥ 150 mg/dl  yes  no | n = 22 (27.5%)  n = 58 (72.5%) | n = 19 (22.4%)  n = 66 (77.6%) | χ2 = 0.59 | 1.32 [0.65, 2.68] | .444 |
| HDL cholesterol < 50 (♀) or  < 40 (♂) mg/dl  yes  no | n = 31 (38.8%)  n = 49 (61.3%) | n = 8 (9.4%)  n = 77 (90.6%) | χ2 = 19.65 | 6.09 [2.59; 14.33] | **< .001** |
| Glucose ≥ 100 mg/dl  yes  no | n = 7 (8.8%)  n = 73 (91.3%) | n = 12 (14.1%)  n = 73 (85.9%) | χ2 = 1.17 | 0.58 [0.22; 1.57] | .280 |

**Table S4** Proportions and statistics for MetS and MetS components, depending on sex in the total cohort.

ADHD: Attention-deficit/hyperactivity disorder, HC: Healthy controls, OR: odds ratio, CI: confidence

interval, MetS: Metabolic syndrome, RR sys: Systolic blood pressure, RR dia: Diastolic blood pressure

| **Variable** | **ADHD** | **HC** | **statistics** | **Cohens’s D** | **p value** |
| --- | --- | --- | --- | --- | --- |
| Waist circumference | 87.3 (14.3) cm | 81.3 (11.6) cm | t_162_ = -2.97 | 0.46 [0.15; 0.77] | **.003** |
| RR sys daytime | 126.8 (12.6) mmHg | 124.3 (9.9) mmHg | t_161_ = -1.37 | 0.21 [-0.10; 0.52] | .175 |
| RR dia daytime | 84.2 (8.6) mmHg | 83.1 (6.7) mmHg | t_161_ = -0.92 | 0.14 [-0.16; 0.45] | .357 |
| Triglycerides | 126.4 (71.6) mg/dl | 122.7 (69.6) mg/dl | t_163_ = -0.33 | 0.05 [-0.25; 0.36] | .738 |
| HDL cholesterol | 60.0 (17.9) mg/dl | 59.4 (17.3) mg/dl | t_163_ = -0.22 | 0.03 [-0.27; 0.34] | .828 |
| Glucose | 90.5 (8.7) mg/dl | 89.9 (7.7) mg/dl | t_163_ = -0.46 | 0.07 [-0.23; 0.38] | .644 |

**Table S5** Comparison of between group differences of dimensional MetS measures.

ADHD: Attention-deficit/hyperactivity disorder, HC: Healthy controls, RR sys: Systolic blood pressure, RR dia: Diastolic blood pressure.

| **Variable** | **ADHD** | **HC** | **statistics** | **Cohens’s D** | **p value** |
| --- | --- | --- | --- | --- | --- |
| Total cholesterol | 182.7 (40.0) mg/dl | 190.6 (40.3) md/dl | t_163_ = -1.28 | -0.20 [-0.50; 0.11] | .204 |
| LDL cholesterol | 108.3 (32.9) mg/dl | 117.0 (32.6) mmHg | t_163_ = -1.69 | -0.26 [-0.57; 0.04] | .093 |
| CRP | 0.16 (0.30) md/dl | 0.14 (0.16) md/dl | t_163_ = 0.42 | 0.07 [-0.24; 0.37] | .675 |
| IL-6 | 1.62 (1.63) mg/dl | 1.73 (1.44) mg/dl | t_163_ = -0.46 | -0.07 [-0.38; 0.23] | .647 |

**Table S6** Comparison of between group differences for further blood lipids and inflammatory markers.

ADHD: Attention-deficit/hyperactivity disorder, HC: Healthy controls, CRP: C-reactive protein, IL-6: Interleukin-6

| **Variable** | **ADHD** | **HC** | **statistics** | **Cohens’s D/OR**  **[95% CI]** | **p value** |
| --- | --- | --- | --- | --- | --- |
| Systolic 24 hours | M = 119.7 (12.1) mmHg | M = 116.6 (9.04) mmHg | t_161_ = 1.853 | 0.29 [-0.02; 0.60] | .066 |
| Systolic RR daytime | M = 126.8 (12.6) mmHg | M = 124.3 (9.9) mmHg | t_161_ = 1.37 | 0.22 [-0.10; 0.52] | .173 |
| Systolic RR nighttime | M = 108.1 (13.9) mmHg | M = 103.8 (10.3) mmHg | t_161_ = 2.21 | 0.35 [0.04; 0.66] | **.029** |
| Systolic nighttime dipping | M =14.8 (6.0)% | M = 16.4 (5.5)% | t_160_ = -2.21 | -0.28 [-0.59; 0.03] | .078 |
| Systolic dipping < 10% | n = 16 (19.5%) | n = 10 (12.5%) | χ2 = 1.48 | 1.70 [0.72; 4.01] | .224 |
| Diastolic 24 hours | M = 78.3 (7.8) mmHg | M = 76.7 (5.9) mmHg | t_161_ = 1.53 | 0.24 [-0.07; 0.55] | .129 |
| Diastolic RR daytime | M = 84.2 (8.6) mmHg | M = 83.1 (6.7) mmHg | t_161_ = 0.92 | 0.14 [-0.16; 0.45] | .357 |
| Diastolic RR nighttime | M =68.6 (8.3) mmHg | M = 66.5 (6.5) mmHg | t_161_ = 1.74 | 0.27 [-0.04; 0.58] | .085 |
| Diastolic nighttime dipping | M = 18.5 (6.3)% | M = 19.8 (6.0)% | t_160_ = -1.34 | -0.21 [-0.52; 0.10] | .183 |
| Diastolic dipping < 10% | n = 9 (11.0%) | n = 2 (2.5%) | χ2 = 4.60 | 4.81 [1.01; 23.00] | **.032** |
| Heart rate 24 hours | M = 72.0 (8.9) min^-1^ | M = 69.3 (8.4) min^-1^ | t_161_ = 1.98 | 0.31 [0.00; 0.62] | **.050** |
| Heart rate daytime | M = 77.7 (9.8) min^-1^ | M = 75.2 (9.6) min^-1^ | t_161_ = 1.68 | 0.26 [-0.10; 0.52] | .095 |
| Heart rate nighttime | M = 62.2 (9.1) min^-1^ | M = 60.5 (8.8) min^-1^ | t_161_ = 1.22 | 0.19 [-0.12; 0.50] | .225 |
| Heart rate dipping | M = 19.9 (7.5)% | M = 19.0 (10.2) min^-1^ | t_160_ = 0.64 | 0.10 [-0.21; 0.41] | .522 |
| Heart rate dipping < 10% | n = 8 (9.8%) | n = 14 (17.5%) | χ2 = 1.48 | 0.51 [0.20; 1.29] | .150 |

**Table S7** Mean systolic, diastolic and heart rate values for 24 hours, daytime, and nighttime. Nighttime

dipping: Mean difference between daytime and nighttime values in percent. Dipping < 10%: Proportion of

participants with less than 10% day- to nighttime dipping. ADHD: Attention-deficit/hyperactivity disorder,

HC: Healthy controls, OR: odds ratio, CI: confidence interval
